# Early Detection of Cerebral Vasospasm Following Aneurysmal Subarachnoid Hemorrhage by IL-6 Electrochemiluminescence Analysis of the Cerebrospinal Fluid

**DOI:** 10.3390/diagnostics16111690

**Published:** 2026-05-30

**Authors:** Adi Ahmetspahic, Ema Selimovic, Faruk Alagic, Almaida Alagic, Ermin Begovic, Suzana Tihic, Almir Dzurlic, Razim Mahmutagic, Mirza Pojskic, Alberto Feletti

**Affiliations:** 1 Department of Neurosurgery, Clinical Center of University of Sarajevo, 71000 Sarajevo, Bosnia and Herzegovina; adi.ahmetspahic@ssst.edu.ba (A.A.); almir.dzurlic@ssst.edu.ba (A.D.); 2 Medical School, Sarajevo School of Science and Technology, 71000 Sarajevo, Bosnia and Herzegovina; ema.selimovic@stu.ssst.edu.ba; 3 Faculty of Pharmacy, University of Sarajevo, 71000 Sarajevo, Bosnia and Herzegovina; faruk.alagic@outlook.com (F.A.); almaida.alagic@outlook.com (A.A.); 4 Department of Neurosurgery, Cantonal Hospital “Dr. Irfan Ljubijankić”, 77000 Bihać, Bosnia and Herzegovina; 5 Organizational Unit Clinical Biochemistry and Immunology, Clinical Center of University of Sarajevo, 71000 Sarajevo, Bosnia and Herzegovina; begovic.ermin@hotmail.com (E.B.); suzanakapidzic@yahoo.com (S.T.); 6 Faculty of Medicine, University of Sarajevo, 71000 Sarajevo, Bosnia and Herzegovina; razim_m@outlook.com; 7 Department of Neurosurgery, Philipps University of Marburg, 35043 Marburg, Germany; 8 Department of Neurosciences, Biomedicine and Movement Sciences, Institute of Neurosurgery, University of Verona, 37126 Verona, Italy; alberto.feletti@univr.it

**Keywords:** subarachnoid hemorrhage, neuroinflammation, cerebral vasospasm, cerebrospinal fluid, interleukin-6, electrochemiluminescence immunoassay

## Abstract

**Background/objectives:** Aneurysmal subarachnoid hemorrhage (aSAH) is a neurosurgical emergency associated with cerebral vasospasm, representing an important complication. This study assessed the association between cerebrospinal fluid (CSF) interleukin-6 (IL-6) levels and cerebral vasospasm after aSAH. **Methods:** This prospective single-center observational study with repeated measurements included 56 patients among 74 screened patients with aSAH; 18 patients were excluded according to predefined exclusion criteria, including infection, unavailable CSF samples or requirement for CSF diversion within the first seven days of onset. The samples were obtained via lumbar puncture on post-hemorrhage days 3 and 7. IL-6 was quantified using an electrochemiluminescent immunoassay. Radiological vasospasm (RV) was assessed by computed tomography angiography, while clinical vasospasm (CCV) was defined as new neurological deterioration unexplained by other causes. **Results:** Median IL-6 levels were 423.5 pg/mL on day 3 and 726.0 pg/mL on day 7. Day 7 IL-6 levels were associated with RV (*p* = 0.045) and CCV (*p* = 0.010), while day 3 IL-6 was associated with CCV only (*p* = 0.035). Day 7 IL-6 showed modest discriminatory performance for CCV: AUC = 0.722, cut-off 460 pg/mL, sensitivity 75.0%, and specificity 68.8%; for RV: AUC = 0.659, cut-off 451 pg/mL, sensitivity 81.8%, and specificity 56.5%. **Conclusions:** Elevated CSF IL-6 levels were associated with cerebral vasospasm after aSAH, with a more consistent association observed for CCV than for RV; however, these findings are hypothesis-generating and require validation before clinical applicability can be determined.

## 1. Introduction

Aneurysmal subarachnoid hemorrhage (aSAH) is a leading cause of cerebrovascular morbidity, with a high mortality rate. De Rooij et al. [1] reported an overall incidence of approximately 9 cases per 100,000 person-years, with significant regional variation. The development of secondary complications following aSAH, particularly cerebral vasospasm, is an important determinant of clinical outcome. Cerebral vasospasm may manifest as radiological vasospasm (RV), defined by angiographic imaging-based vessel narrowing, or as clinical cerebral vasospasm (CCV), characterized by new neurological deficits. Interleukin-6 (IL-6) is a primary neuroinflammatory mediator in nervous tissue [2]. This proinflammatory cytokine is secreted by mononuclear phagocytes, T cells, and endothelial cells in response to aSAH [3]. Vlachogiannis et al. [4] reported inflammatory response within the intrathecal compartment—specifically the subarachnoid space—shortly after the initial hemorrhage. This finding suggested endogenous cytokine production within the central nervous system (CNS), independent of systemic inflammation. This distinction may be clinically relevant, as the presence of blood and its breakdown products within the subarachnoid space may contribute to local neuroinflammatory cascades, resulting in a compartment-specific inflammatory response that could be relevant to vasospasm pathophysiology; however, the clinical significance of cerebrospinal fluid (CSF) measurements compared with blood-based markers remains to be established. Consistent with this, even in patients with unruptured intracranial aneurysms (UIA), CSF IL-6 concentrations have been shown to exceed plasma levels despite an intact blood–brain barrier, supporting local cytokine synthesis within the CNS [5].

Experimental and preclinical studies have demonstrated that inflammatory pathways contribute to early brain injury following aSAH [6,7,8,9]. Several clinical studies have also reported associations between elevated CSF IL-6 levels and vasospasm, delayed cerebral ischemia, disease severity, and clinical outcomes after aSAH [5,10,11,12,13,14,15]; however, published findings remain heterogeneous due to differences in sampling methods, timing of measurements, biological matrices, assay techniques, and outcome definitions [3,16]. Importantly, most studies have not separately analyzed CCV and RV despite their incomplete overlap and distinct implications. Additionally, CSF IL-6 measurement using electrochemiluminescence immunoassay (ECLIA) remains insufficiently evaluated in this context, with most existing literature relying on enzyme-linked immunosorbent assay (ELISA)-based approaches. Although CSF sampling may provide insight into compartment-specific inflammatory dynamics, lumbar puncture (LP) entails procedural burden and cannot be performed in all patients, particularly those with obstructive hydrocephalus or contraindications to the procedure. Therefore, whether CSF IL-6 provides clinically meaningful incremental information beyond peripheral blood IL-6 for vasospasm-related assessment remains uncertain and requires further investigation [17,18].

IL-6 levels rise early after hemorrhage, with initial increases within the first 72 h and peak values typically observed within the first week [19,20]. This temporal pattern precedes or overlaps with the recognized period of increased vasospasm risk. Accordingly, the first week after aSAH appears to correspond to a period of increased susceptibility to vasospasm, supporting the rationale for early serial CSF sampling.

This study aimed to evaluate serial CSF IL-6 levels after aSAH and examine their association with CCV and RV using the ECLIA method.

## 2. Methods

The research design was a prospective single-center observational exploratory investigation conducted at the Department of Neurosurgery of the Clinical Center of the University of Sarajevo (CCUS) between 2022 and 2024. CSF analysis was performed in the Organizational Unit Clinical Biochemistry and Immunology of the CCUS. During the study period, 74 patients had verified aSAH, of whom 18 were excluded according to predefined criteria. The final cohort consisted of 56 patients eligible for serial LP-based CSF sampling on post-hemorrhage days 3 and 7, resulting in a total of 112 CSF samples collected for analysis. The inclusion criteria were as follows: patients who were older than 18 years, had radiologically confirmed aSAH, and had been admitted to the hospital within 48 h of the onset of aSAH. Conversely, the exclusion criteria were more stringent and included: patients who were younger than 18 years of age, those who had undergone cardiopulmonary resuscitation, cardiac arrest, or had not responded to brainstem reflex stimulation, as determined by the Glasgow Coma Scale (GCS). Additionally, patients with bilateral mydriasis, non-occluded aneurysms, external ventricular drain (EVD) placement, lumbar drainage, or cistern drainage at any point during the first seven days, patients who had not undergone a second measurement of CSF values for any reason, including lethal outcome, transfer to another institution, or patients in whom sampling methods were not feasible, coagulation of the samples (defined as spontaneous clot formation rendering the sample unsuitable for pipetting and reliable IL-6 analysis), febrile conditions, systemic inflammatory diseases, meningitis or any other clinical or laboratory evidence of CNS infection at the time of LP sampling, renal insufficiency, liver failure, hematological and oncological diseases, patients who had been receiving corticosteroid therapy, neurodegenerative diseases, patients who had been receiving Biotin therapy, a history of COVID-19 infection within the preceding three months, and patients with prior LP before study enrollment. The present analysis is restricted to a clinically selected subgroup of aSAH patients in whom (i) no early continuous CSF diversion was required and (ii) serial LP was both safe and feasible. Patients with acute symptomatic hydrocephalus, severe intracranial hypertension, or other indications for early EVD or lumbar/cisternal drainage within 7 days of onset are not represented in this cohort.

**Figure 1:**
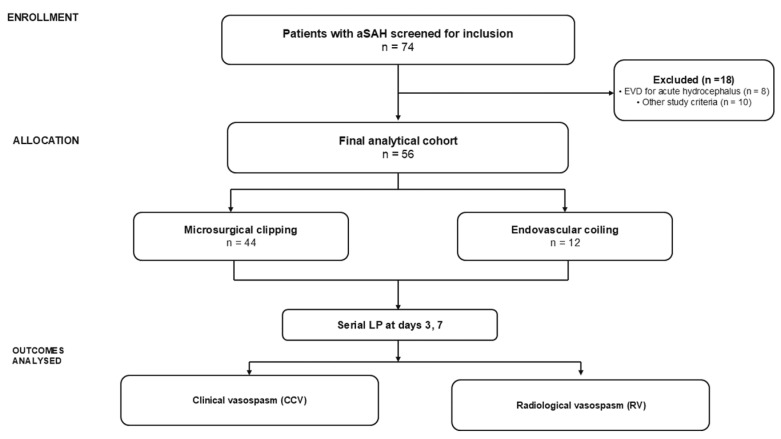
Cohort selection flow for the CSF IL-6 and cerebral vasospasm analysis. Of 74 patients with aSAH screened during the study period, 18 were excluded: 8 required EVD for acute hydrocephalus (incompatible with the study protocol); 10 met other prespecified study exclusion criteria. The final analytical cohort consisted of 56 patients, who underwent serial LP on days 3 and 7 and were subsequently analyzed for the two predefined endpoints—CCV and RV.

The research was conducted in accordance with the fundamental principles of the most recent edition of the Declaration of Helsinki (2013) and pertains to the rights of patients involved in biomedical research, and was approved by the Institutional Ethics Committee with registration number 06-04-9-29298. The variables considered included age, sex, World Federation of Neurosurgical Societies (WFNS) grade, Hunt and Hess grade, modified Fisher score, GCS score, and treatment modality (surgical clipping vs. endovascular coiling). Patient enrollment, CSF sampling at predefined time points (days 3 and 7), and IL-6 analysis were performed prospectively, whereas clinical and radiological outcomes were retrospectively extracted from medical records, discharge summaries, and radiology reports. These variables were analyzed in relation to CSF IL-6 levels at predefined measurement time points and CCV and RV, using univariate analysis; however, no multivariable adjustment was performed because of sample size limitations. A detailed flow diagram of the patient selection process is presented in Figure 1.

### 2.1. Outcome Variables

CCV was defined in accordance with established consensus definitions proposed by Vergouwen et al. [21] as documented new focal neurological deficits or a decrease of ≥2 points on the GCS not attributable to other causes. RV was defined as a diffuse reduction in the caliber of the major intracranial vessels, corresponding to an arterial diameter reduction of ≥25% on follow-up computed tomography angiography (CTA) compared with the baseline vessel caliber documented on admission CTA [22]. Radiological assessments were used as documented in official reports; inter-rater reliability was not formally assessed, and no adjudication of discordant findings was performed. Outcome variable assessments were conducted based on clinical indication rather than at fixed time points, across a standardized follow-up period of up to 21 days post-aSAH [23], with CSF IL-6 levels examined in relation to vasospasm status determined during this interval.

### 2.2. Sampling Methods

In the present study, CSF was sampled via serial LP after the aneurysm had been secured by ultra-early treatment [24]. In keeping with the institutional standard of care, LP was selected as a recognized alternative to preventive continuous CSF diversion in selected aSAH patients, routinely performed for both therapeutic and diagnostic purposes and associated with a lower infection risk compared with indwelling CSF diversion devices [25,26,27,28,29]; CSF analysis for IL-6 analysis was conducted as part of this established clinical procedure. However, in patients in whom acute hydrocephalus was suspected on clinical and radiological grounds at any time during the first seven days, CSF diversion (EVD) was placed without delay, and these patients were excluded from the study. The same approach was applied uniformly to patients managed by microsurgical clipping and endovascular coiling, with no differences in LP feasibility or timing between the two groups. In both groups, no routine intraoperative or postoperative cisternal drainage [30,31,32] or periprocedural CSF diversion was performed as standard practice [33,34]. Intraoperative cisternal opening or fenestration during aneurysm surgery was not classified as cisternal drainage unless an indwelling cisternal drain was left in place. No included patient had an indwelling cisternal drain during the predefined CSF sampling interval. Patients from the screened population who required EVD, lumbar drainage, or cisternal drainage within the first seven days were excluded from the analytical cohort. Standard CSF analysis at each LP sampling time point included biochemical, cytological, and microbiological parameters, which together provided routine screening for potential signs of CNS infection during the sampling interval, in addition to their analytical role. For each LP, three mL of CSF was collected aseptically. To minimise the potential effect of blood contamination, the initial CSF outflow was discarded; approximately 10 mL was allowed to drain until a visually clear specimen was obtained, after which the sample was collected and submitted for laboratory analysis. Patients who required CSF diversion after the sampling interval were permitted to undergo such procedures on clinical grounds and were retained in the cohort, as their CSF sampling series had been completed prior to any diversion.

### 2.3. Laboratory Setup

All samples were analyzed within 30 min of sampling. Prior to analysis, samples underwent routine pre-analytical handling according to institutional procedures and manufacturer recommendations. CSF samples were centrifuged at 2000× *g* for 10 min to sediment erythrocytes and reduce their potential influence on IL-6 quantification. The supernatant was subsequently used for analysis. Given that all patients had confirmed aSAH, the presence of blood in CSF was considered an inherent feature of the disease rather than a pre-analytical contaminant. The samples were analyzed using the in vitro immunoassay kit for quantitative determination of IL-6 in humans, ECLIA on the Cobas Pro e801 analytical unit (Roche Diagnostics GmbH, Mannheim, Germany). The assay is validated by the manufacturer for serum and plasma samples; therefore, its use in CSF represents an off-label application. The manufacturer’s instructions were strictly adhered to during testing procedures with a total immunoassay duration of 18 min. Samples with visible blood contamination were interpreted with caution in the context of clinical findings. The analytical detection range of the assay was 1.5–5000 pg/mL. In cases where IL-6 concentrations exceeded the upper detection limit, the analyzer performed automatic redilution according to its built-in protocol, with results corrected for the dilution factor. Overall, 7 of 112 CSF samples (6.25%) exceeded the upper detection limit and required automatic redilution by the analyzer, including 5 samples (4.46%) from day 3 and 2 samples (1.78%) from day 7. The remaining 105 samples (93.75%) were measured within the analytical range without dilution. Repeated measurements were performed only when flagged by the analyzer (e.g., carry-over errors), in accordance with standard instrument protocols. No manual repeat measurements were performed outside these procedures. The sample analysis results were obtained within one hour of sampling. The values were tabulated as absolute numbers under the corresponding code for each respondent.

### 2.4. Statistical Analysis

Statistical analyses were performed using IBM SPSS Statistics, version 27.0 (IBM Corp., Armonk, NY, USA). Continuous variables were assessed for normality. Non-normally distributed continuous variables were compared between independent groups using the Mann–Whitney U test, while paired comparisons (day 3 vs. day 7) were performed using the Wilcoxon signed-rank test. Categorical variables were analyzed using the chi-square test or Fisher’s exact test, as appropriate. Baseline clinical and radiological severity variables, including WFNS grade, Hunt and Hess grade, modified Fisher grade, GCS score, age, and treatment modality, were assessed in univariable analyses. No multivariable adjustment was performed because of the limited sample size and the risk of model overfitting. The diagnostic performance of IL-6 in relation to CCV and RV was evaluated using receiver operating characteristic (ROC) curve analysis. The area under the curve (AUC) was calculated as a measure of discriminatory performance, with corresponding 95% confidence intervals (CIs). Optimal cut-off values were determined using the Youden index. These cut-off values were internally derived and should be interpreted as exploratory thresholds rather than clinically established values. Given the limited sample size and the absence of multivariable adjustment, all association and ROC-based analyses should be interpreted as exploratory and hypothesis-generating. *p*-value < 0.05 was considered statistically significant.

## 3. Results

A total of 56 patients were included, comprising 16 (28.6%) males and 40 (71.4%) females. Of the total cohort, 44 patients (78.6%) underwent surgical clipping and 12 patients (21.4%) underwent endovascular treatment. No statistically significant difference in CSF IL-6 levels was detected between the clipping and endovascular treatment groups on day 3 (*p* = 0.618) or day 7 (*p* = 0.168). Given the marked imbalance in group sizes (44 clipping vs. 12 coiling patients), this comparison is underpowered, and the absence of a statistically significant difference cannot be interpreted as evidence of equivalence between the two treatment modalities. The baseline demographic and clinical characteristics of the study cohort are summarized in Table 1.

Baseline clinical and radiological severity variables were assessed for association with vasospasm outcomes in exploratory univariable analyses. Continuous variables were categorized into clinically relevant groups: age (1–30, 31–50, 51–65, >65 years) and GCS (13–15, 8–12, 5–7, <5). All analyses were performed using Fisher’s exact test.

No statistically significant associations were detected between WFNS grade and CCV (*p* = 0.224) or RV (*p* = 0.813); between GCS and CCV (*p* = 0.339) or RV (*p* = 0.491); between age and CCV (*p* = 0.413) or RV (*p* = 0.189); between Hunt and Hess grade and CCV (*p* = 0.159) or RV (*p* = 0.702); or between modified Fisher grade and CCV (*p* = 0.117) or RV (*p* = 0.537). Given the limited sample size and the categorization of continuous variables, these univariable analyses were not powered to reliably detect modest associations and are reported descriptively.

Across the sampling interval (post-haemorrhage days 3 and 7), routine cytological and microbiological screening of CSF samples did not reveal signs of CNS infection in any patient. The median CSF IL-6 level was 423.5 pg/mL (interquartile range [IQR], 127.3–3120.3; range, 7.0–21,248.0) on day 3 and 726.0 pg/mL (IQR, 196.0–1998.0; range, 6.0–25,841.0) on day 7. However, this difference was not statistically significant in paired analysis (Wilcoxon signed-rank test, *p* = 0.087).

Patients with CCV had higher CSF IL-6 levels at both time points, with larger effect sizes observed on day 7. For RV, the difference was not statistically significant on day 3 but reached statistical significance on day 7 (Table 2).

CCV was present in 40 patients (71.4%) and absent in 16 patients (28.6%). In contrast, RV was observed in 33 patients (58.9%) and was absent in 23 patients (41.1%). The overlap and discordance between CCV and RV are summarized in Table 3.

Cross-tabulation analysis showed that 51.8% of participants were positive for both CCV and RV, while 7.1% were RV-positive but CCV-negative. Furthermore, 19.6% of participants were RV-negative but CCV-positive, whereas 21.4% were negative on both tests. Overall, discordant CCV and RV findings were observed in 26.8%, contributing to the higher overall prevalence of CCV (71.4%) compared to RV (58.9%). A statistically significant association between CCV and RV findings was observed (χ^2^ = 10.654; df = 1; *p* = 0.001). However, the presence of discordant cases indicates that CCV and RV were not fully interchangeable outcomes in this cohort.

**Figure 2:**
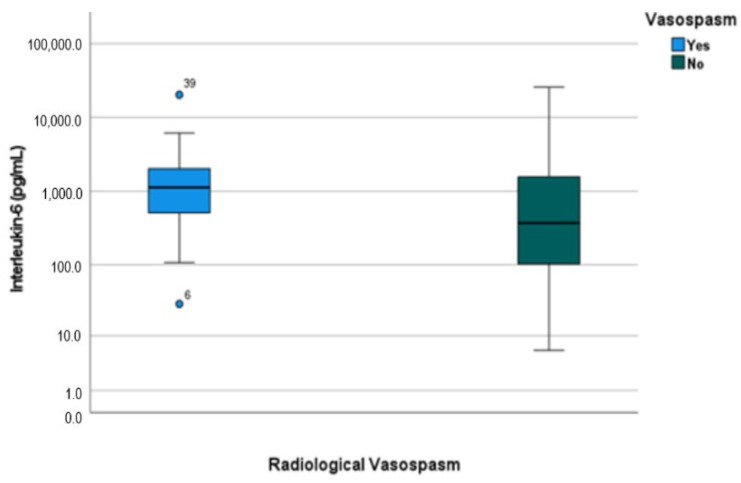
Comparison of Day 7 IL-6 levels by RV. Note: The vertical axis in Figure 2 is presented on a logarithmic scale due to the wide range of IL-6 values spanning several orders of magnitude. This approach was used to improve visualization and allow clearer presentation of data distribution.

Day 7 CSF IL-6 levels showed a borderline statistically significant association with RV (*p* = 0.045), whereas day 3 levels were not significantly associated with RV (*p* = 0.075), as illustrated in Figure 2.

**Figure 3:**
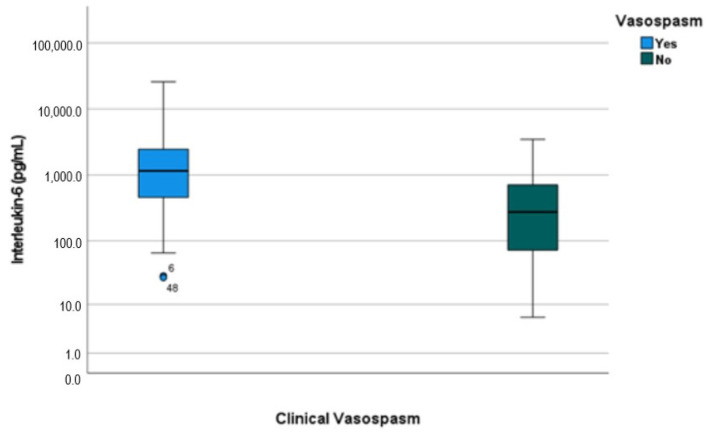
Comparison of Day 7 IL-6 levels by CCV. Note: The vertical axis in Figure 3 is presented on a logarithmic scale due to the wide range of IL-6 values spanning several orders of magnitude.

Day 7 IL-6 levels were associated with CCV (*p* = 0.01), as illustrated by the boxplots in Figure 3. Notably, IL-6 levels on day 3 also showed a significant association with CCV (*p* = 0.035).

**Figure 4:**
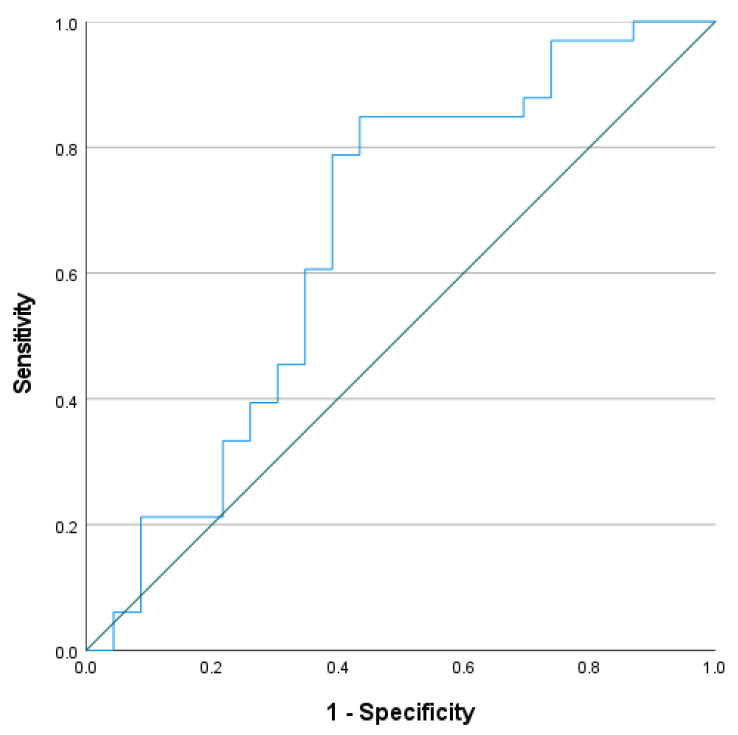
ROC analysis of day 7 IL-6 levels for RV. The blue curve represents the ROC curve, whereas the green diagonal line indicates no discriminative ability (AUC = 0.5).

ROC analysis of day 7 CSF IL-6 levels in relation to RV showed modest discriminatory performance. The AUC was 0.659 (95% CI: 0.504–0.814; *p* = 0.045). The internally derived cutoff value, determined by the Youden index (0.383), was 451 pg/mL, corresponding to a sensitivity of 81.8% and a specificity of 56.5%. The positive and negative likelihood ratios were 1.88 and 0.32, respectively. These findings are illustrated in Figure 4.

**Figure 5:**
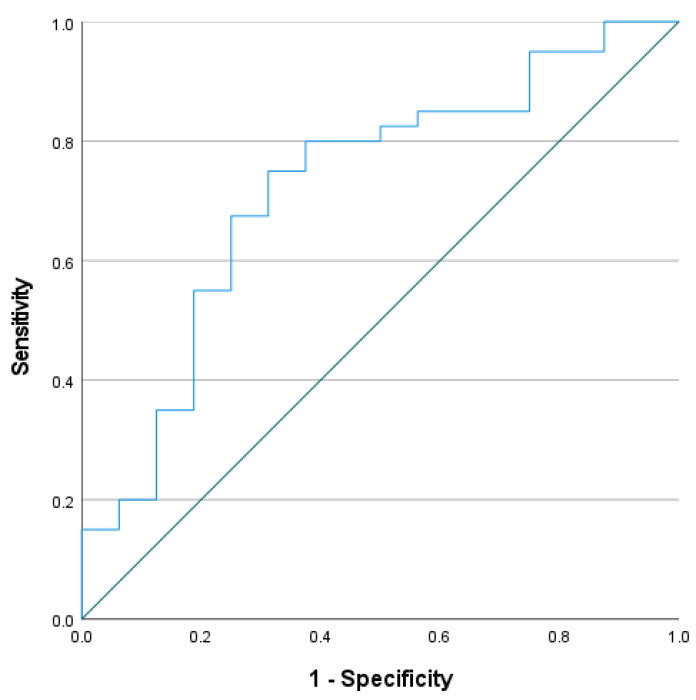
ROC analysis for IL-6 on day 7 in relation to CCV. The blue curve represents the ROC curve, whereas the green diagonal line indicates no discriminative ability (AUC = 0.5).

ROC analysis of day 7 CSF IL-6 levels in relation to CCV showed moderate discriminatory performance. The AUC was 0.722 (95% CI: 0.57–0.874; *p* = 0.01). The internally derived cutoff value, determined by the Youden index (0.438), was 460 pg/mL, corresponding to a sensitivity of 75.0% and a specificity of 68.8%. The positive and negative likelihood ratios were 2.40 and 0.36, respectively. These findings are illustrated in Figure 5.

Statistical comparison of the ROC curves is presented in Table 4.

## 4. Discussion

In this exploratory study, elevated CSF IL-6 levels in the early post-hemorrhagic period were associated with cerebral vasospasm following aSAH. On post-hemorrhage day 7, IL-6 concentrations were associated with vasospasm across both CCV and RV definitions, whereas day 3 levels were associated only with clinically defined vasospasm. Given the single-centre design, limited sample size, internally derived thresholds, and the only modest discriminatory performance observed on ROC analysis, CSF IL-6 should be regarded as a potential exploratory biomarker rather than a validated predictive marker. Because only CSF IL-6 was measured, the present findings may reflect compartment-specific CNS inflammatory activity and do not permit inferences regarding systemic inflammation. Several methodological considerations reinforce this cautious interpretation. The lack of significant associations between severity-related variables and vasospasm outcomes should not be taken as evidence that disease severity does not influence the outcome; with the present sample size and the categorization of continuous variables, these exploratory analyses were not powered to detect modest effects, and residual confounding by severity cannot be excluded. Likewise, the absence of a detected difference between clipping and coiling does not imply equivalence, as this comparison is markedly underpowered owing to the imbalance in group sizes (44 vs. 12). These findings should therefore be regarded as hypothesis-generating and require confirmation in larger, more balanced cohorts.

CCV and RV were associated with one another but did not fully overlap: a substantial proportion of patients exhibited discordant findings, with some showing CCV in the absence of RV and vice versa. This partial dissociation is consistent with the higher observed prevalence of CCV (71.4%) compared with RV (58.9%) and aligns with Haq et al. [14], who reported that IL-6 combined with endothelin-1 was associated with early vasospasm, including cases without radiological correlates. Because CCV is defined by the development of neurological symptoms while RV relies on imaging assessment (CTA in our cohort), the two constructs may capture overlapping but not identical pathophysiological phenomena. The association observed between day 3 IL-6 and CCV, but not RV, may be compatible with early inflammatory activity preceding radiographically detectable vessel changes; however, it may equally reflect differences in outcome definition, the timing of clinical and radiological assessments, or limited sample size. Because the present data do not allow these alternatives to be distinguished, this observation should be regarded as hypothesis-generating rather than as evidence of a defined temporal sequence between inflammatory activation and structural vasospasm.

Our observations are compatible with the broader concept that intrathecal inflammation may contribute to vasospasm pathophysiology following aSAH, although the present study does not establish a direct mechanistic link. This interpretation is consistent with the findings of Kamińska et al. [35], who investigated IL-6 concentrations in both plasma and CSF using a comparable analytical approach, albeit on a different analyser platform, in the context of unruptured intracranial aneurysms; their data supported the possibility of intrathecal IL-6 production independent of systemic circulation, even in the presence of an intact blood–brain barrier. Further supporting compartmental dissociation, Bandyopadhyay et al. [15] reported divergent kinetics between plasma and CSF IL-6, with plasma concentrations peaking earlier and CSF levels remaining elevated through the subacute phase; that study incorporated a broader cytokine panel and combined EVD- and LP-based CSF sampling with parallel plasma measurements, which limits direct comparability with the present findings.

Within the broader literature, recent work continues to support the relevance of CSF IL-6 in aSAH while highlighting substantial methodological heterogeneity. A systematic meta-analysis by Croci et al. [16] identified an association between elevated plasma and CSF IL-6 levels and the occurrence of radiological vasospasm, although interpretation is limited by the potential influence of acute-phase complications on circulating IL-6. In a larger cohort, Ketelauri et al. [12] reported that CSF IL-6 levels during the first week following aSAH were associated with in-hospital mortality and unfavourable 6-month outcomes; notably, patients developing radiological vasospasm exhibited a more pronounced inter-interval rise between days 1–3 and days 4–6, suggesting that temporal IL-6 dynamics may warrant further evaluation in prognostic models. Similarly, Onda et al. [13] demonstrated that lower postoperative CSF IL-6 levels were associated with more favourable outcomes and that early postoperative IL-6 differed according to vasospasm status; that study, however, was restricted to surgically clipped patients and therefore reflects a more selected clinical context than the present mixed cohort.

### 4.1. CSF IL-6 Cut-Off Values for Cerebral Vasospasm After aSAH

Although ECLIA-measured CSF IL-6 levels have been reported in prior studies, no universally standardised reference ranges have been established, in part owing to the ethical constraints associated with obtaining CSF from healthy individuals. The Cobas Pro e801 analyser (Roche Diagnostics) employed in the present study detects IL-6 concentrations between 1.5 and 5000 pg/mL; while the plasma reference interval is defined as 7 pg/mL (95th percentile), no equivalent CSF reference interval is currently available.

Against this background, the present findings provide preliminary exploratory evidence of an association between ECLIA-measured CSF IL-6 and cerebral vasospasm. These observations may reflect biological variability in IL-6 expression, heterogeneity in the timing of vasospasm onset, and the use of single time-point measurements. The relatively small sample size and internal derivation of cut-off values may have introduced variability and potential overfitting. The exploratory cut-off identified in the range of approximately 450–460 pg/mL is therefore subject to optimism bias and should not be applied in clinical practice without external validation in independent cohorts; it is presented as a preliminary observation to inform future prospective studies rather than as a clinically actionable threshold. The absence of significant associations between baseline severity variables and vasospasm outcomes should likewise be interpreted cautiously, as the study was not powered to exclude residual confounding by initial disease severity.

Cross-study comparisons should be approached with caution given the substantial methodological and clinical heterogeneity across the existing literature, including differences in assay platforms, patient selection, disease severity, sampling timing, outcome definitions, and analytical approaches. Ni et al. [11] identified a cut-off of approximately 400 pg/mL associated with vasospasm within the first seven days after aSAH; Lenski et al. [5] reported a threshold of 530 pg/mL for CCV, although interpretation was complicated by overlapping IL-6 elevations in concurrent ventriculitis; Yao et al. [36] reported a lower threshold of 247.89 pg/mL in severe aSAH. These comparisons are further complicated by prior studies frequently employing EVD-based or mixed EVD/LP sampling rather than a uniform LP-based approach. Variability in reported thresholds may additionally reflect differences in outcome definitions and diagnostic modalities; several studies have not distinguished between CCV and RV or have relied on digital subtraction angiography or transcranial Doppler, whereas the present study applied a CTA-based definition. Although recent contributions by Haq et al. [14] and Ketelauri et al. [12] offer additional clinical insight, comparable differences in study design, sampling strategy, and analytical approach similarly limit direct comparison.

### 4.2. Methodological Considerations of LP-Based ECLIA CSF IL-6 Measurement in aSAH

The serial LP-based CSF management adopted in this study reflects an established alternative to continuous CSF diversion in selected aSAH patients and carries a lower procedural infection risk than indwelling devices [26,27,28]. Recent comparative evidence supports LP as an initial CSF management option in patients without acute symptomatic hydrocephalus, with comparable outcomes and fewer device-related complications relative to EVD or continuous lumbar drainage [25,29]. The absence of routine periprocedural cisternal drainage in the clipping group is methodologically supported by our institutional standard of intraoperative cisternal opening with routine tandem fenestration of the lamina terminalis and the membrane of Liliequist, manoeuvres consistently associated with a reduced incidence of shunt-dependent hydrocephalus [30,31,32]. Likewise, the absence of routine CSF diversion in the coiling group is consistent with AHA/ASA recommendations, which reserve continuous diversion for acute symptomatic hydrocephalus rather than as a routine endovascular adjunct [34]; selective reactive placement, including in cases of intraprocedural rerupture identified as angiographic contrast extravasation, is further supported by empirical evidence [33].

Of particular relevance to the present biomarker study, continuous CSF diversion devices carry a recognised risk of device-associated infection [26,27,28], itself a potent stimulus for marked CSF IL-6 elevation independent of vasospasm-related neuroinflammation [5,37,38,39,40]. Intrathecal IL-6 elevation has been shown to precede the clinical diagnosis of EVD-related infection by approximately one day [39], and ventriculitis-associated CSF IL-6 can reach values several-fold above those typically observed in uncomplicated aSAH [38]. Within our sampling window on post-haemorrhage days 3 and 7, which overlaps with the early period during which device-associated infection commonly develops and may produce subclinical IL-6 elevation, inclusion of patients with indwelling drains could have generated biomarker signal that mimicked or amplified vasospasm-related elevation. The exclusive use of serial LP, together with explicit exclusion of patients with clinical or laboratory evidence of CNS infection at sampling, accordingly minimised this specific source of confounding. Although infection-related outcomes were not a primary endpoint, routine cytological and microbiological screening of CSF on days 3 and 7 served as an integral methodological control and revealed no signs of CNS infection during the sampling interval, supporting that the observed IL-6 dynamics most likely reflect vasospasm-related rather than infection-related pathways. The present approach therefore represents an evidence-supported institutional strategy within the contemporary spectrum of aSAH management at our centre rather than an institutional outlier, while inherently restricting the analysed cohort to patients in whom serial LP is feasible, a selection effect addressed separately in the Section 5, Limitations.

Beyond the sampling approach, the analytical platform used for IL-6 quantification also warrants methodological consideration, although the principal contribution of this study lies in the observed association between CSF IL-6 and vasospasm rather than in the platform itself. To our knowledge, no prior study has evaluated an ECLIA-based CSF IL-6 cut-off specifically against CCV as a separate endpoint. As the majority of the existing aSAH literature relies on ELISA-based measurements, direct numerical comparison with previously reported thresholds is inherently limited; absolute IL-6 concentrations obtained by ECLIA and ELISA are not interchangeable, even when the rank ordering of samples is preserved. The Cobas Pro e801 assay is manufacturer-validated for serum and plasma, and its application to CSF therefore constitutes off-label use. Given the distinct biochemical properties of CSF, including lower protein content and differences in matrix composition, potential matrix effects on assay performance cannot be excluded. Prior to study initiation, an internal comparison between ECLIA and ELISA in paired CSF samples yielded comparable results, in addition to in-house assessments of precision, dilution linearity, and limit of detection. Comprehensive CSF-specific validation, particularly formal recovery studies and matrix interference testing, was not performed, and the reported CSF IL-6 concentrations should accordingly be interpreted with caution.

Despite these limitations, ECLIA-based CSF IL-6 measurement has been reported across several neurological contexts. Ridwan et al. [20] documented an association with DCI-related infarctions in aSAH, although this outcome differs from the vasospasm endpoints evaluated in the present study. Kamińska et al. [35] provided evidence of intrathecal IL-6 production using the same platform in patients with intracranial aneurysms, and García-Hernández et al. [40] demonstrated that ECLIA-measured IL-6 may aid in differentiating aseptic from bacterial meningitis. Collectively, these reports support the feasibility of ECLIA-based CSF IL-6 measurement across diverse neurological conditions, while reinforcing the need for cautious interpretation in the absence of formal CSF-specific analytical validation. The ECLIA platform additionally enabled rapid sample processing within the acute-care workflow, which may represent a practical advantage in time-sensitive clinical settings. Collectively, these observations suggest that CSF IL-6 should be interpreted as one component of a broader intrathecal inflammatory profile rather than as an isolated quantitative marker, with its informational value ultimately depending on temporal dynamics and integration with established clinical and radiological assessment.

## 5. Limitations

### 5.1. Several Limitations Warrant Acknowledgement

First, and most importantly, the cohort represents a clinically selected subgroup of patients with aSAH in whom early continuous CSF diversion was not required and serial LP was feasible. Patients with acute hydrocephalus, severe intracranial hypertension, or other indications for early EVD or lumbar/cisternal drainage are therefore not represented, and the findings cannot be generalized to the broader aSAH population, particularly to patients with more severe haemorrhage or early CSF diversion requirements. However, while this selection limits external generalisability, it also reduces analytical confounding from drainage-associated CSF infection, a recognised source of marked IL-6 elevation that is discussed further in the Discussion. The serial LP strategy, described in the Methods, reflects the standard of aSAH care at our centre in the absence of acute hydrocephalus; consequently, direct comparison with cohorts managed under continuous CSF diversion protocols is not possible from these data.

Second, the single-centre design and the limited, markedly imbalanced sample (44 clipping vs. 12 coiling) constrain generalisability and statistical power. The between-modality comparison is underpowered, and the absence of a detected difference should not be interpreted as equivalence; the exploratory univariable analyses of severity-related variables were similarly underpowered, and a true effect of disease severity on the outcomes cannot be excluded.

Third, vasospasm assessments were performed by clinical indication rather than at fixed time points, and RV and clinical data were extracted retrospectively from radiology reports and discharge summaries; this may have affected temporal alignment between IL-6 measurements and vasospasm occurrence, so the observed associations should be interpreted within the observation period rather than as strictly predictive effects.

Fourth, the ECLIA assay is manufacturer-validated for serum and plasma, and its application to CSF constitutes off-label use; although precision, dilution linearity, and limit of detection were assessed, comprehensive CSF-specific validation was not performed, and matrix effects or residual blood-related interference cannot be fully excluded. The absence of standardised CSF IL-6 reference ranges further complicates absolute threshold interpretation.

Finally, the observational, exploratory design precludes causal inference; no multivariable adjustment was performed owing to sample size, and residual confounding by disease severity, treatment modality, assessment timing, or other unmeasured factors cannot be excluded. The absence of a healthy or disease-control group limits external validity, and methodological heterogeneity across the existing literature—including differences in patient selection, outcome definitions, assay platforms, and measurement timing—further restricts direct comparability with prior studies. In particular, because CSF was sampled exclusively via serial LP from the lumbar compartment, direct numerical comparison with prior studies employing EVD-based, continuous lumbar drainage-based, or mixed sampling strategies is inherently limited, and the reported IL-6 concentrations and exploratory thresholds should therefore be interpreted within the context of lumbar CSF obtained by serial LP.

### 5.2. Future Research Directions

The present findings suggest several directions for future research:

Validation in Larger Cohorts: The observed associations require confirmation in larger, multicentre studies with standardized outcome definitions and adequate statistical power for multivariable adjustment.

Comparative Studies: Direct comparison of CSF versus plasma IL-6 in matched cohorts is needed to determine whether compartment-specific sampling provides clinically meaningful incremental information.

Temporal Dynamics: Prospective studies with protocol-driven serial sampling at multiple time points are required to establish the temporal relationship between IL-6 elevation and vasospasm onset.

Integration with Multimodal Assessment: Future work should evaluate whether IL-6 adds predictive value when combined with clinical, radiological, and other biomarker data.

Analytical Validation: CSF-specific validation of ECLIA performance, including matrix effects and interference studies, is required before broader application.

At this stage, future work should focus primarily on validation, temporal characterization, and analytical standardization rather than clinical implementation. At present, there is insufficient evidence to support the use of CSF IL-6 measurements to guide therapeutic decisions, including anti-inflammatory interventions for vasospasm prophylaxis.

## 6. Conclusions

CSF IL-6 levels were associated with cerebral vasospasm following aSAH, with somewhat more consistent findings for CCV than for RV. Given the modest discriminatory performance, limited sample size, and internally derived thresholds, these observations should be regarded as preliminary and hypothesis-generating. Further investigation in larger, prospective, multicentre cohorts is warranted before conclusions regarding the clinical utility of CSF IL-6 in this setting can be drawn.

## Figures and Tables

**Table 1:** Baseline Characteristics of Patients.

Characteristic	Total (*N* = 56)
Sex—no. (%)	
Male	16 (28.6)
Female	40 (71.4)
Treatment—no. (%)	
Surgical clipping	44 (78.6)
Endovascular treatment	12 (21.4)
Radiological Vasospasm—no. (%)	
Present	33 (58.9)
Absent	23 (41.1)
Clinical Vasospasm—no. (%)	
Present	40 (71.4)
Absent	16 (28.6)

**Table 2:** CSF IL-6 levels in patients with aSAH according to CCV and RV status.

Outcome	Time Point	Vasospasm, Median (Q1–Q3)	No Vasospasm Median (Q1–Q3)	*p*-Value	Hodges–Lehmann Difference (95% CI)
CCV	Day 3	654.5 (167.75–3422.75)	164.5 (92.25–446.5)	0.035	266 (15–1347)
CCV	Day 7	1154 (454.5–2497.5)	287 (64.25–784.25)	0.01	598 (105–1358)
RV	Day 3	500 (239.5–3198)	151 (77–3019)	0.075	175 (−18 to 496)
RV	Day 7	1127 (486–2205)	372 (65–1678)	0.045	429 (5–1055)

Values are expressed as median (IQR). Group comparisons were performed using the Mann–Whitney U test. Effect sizes are reported as Hodges–Lehmann estimates with 95% CI. CCV and RV were analyzed separately.

**Table 3:** Cross-tabulation of CCV and RV findings.

	CCV Positive	CCV Negative	Total
RV positive	29	4	33
RV negative	11	12	23
Total	40	16	56

**Table 4:** Comparison of Day 7 IL-6 Discriminatory Performance for CCV vs. RV.

Variable	Radiological Vasospasm	Clinical Vasospasm
Area under the ROC curve	0.659	0.722
*p*-value	0.045	0.01
Optimal cut-off—pg/mL	451	460
Youden index	0.383	0.438
Sensitivity—%	81.8	75.0
Specificity—%	56.5	68.8

## Data Availability

The data in this study are available on request from the first and corresponding author. The data are not publicly available due to privacy restrictions.

## Abbreviations

Abbreviation	Meaning
aSAH	Aneurysmal Subarachnoid Hemorrhage
AUC	Area Under the Curve
CCUS	Clinical Center of the University of Sarajevo
CCV	Clinical Cerebral Vasospasm
CI	Confidence Interval
CNS	Central Nervous System
CSF	Cerebrospinal Fluid
CTA	Computed Tomography Angiography
ECLIA	Electrochemiluminescence Immunoassay
ELISA	Enzyme-Linked Immunosorbent Assay
EVD	External Ventricular Drain
GCS	Glasgow Coma Scale
IL-6	Interleukin-6
IQR	Interquartile Range
LP	Lumbar Puncture
ROC	Receiver Operating Characteristic
RV	Radiological Vasospasm
UIA	Unruptured Intracranial Aneurysm
WFNS	World Federation of Neurosurgical Societies
